# Pseudoangiomatous Stromal Hyperplasia of the Breast: A Rare Finding in a Male Patient

**DOI:** 10.7759/cureus.4923

**Published:** 2019-06-17

**Authors:** Lynsey M Maciolek, Taylor S Harmon, Jing He, Sarfaraz Sadruddin, Quan D Nguyen

**Affiliations:** 1 Radiology, University of Texas Medical Branch, Galveston, USA; 2 Radiology, University of Florida College of Medicine, Jacksonville, USA; 3 Pathology, University of Texas Medical Branch, Galveston, USA

**Keywords:** breast masses, interanastomosing, mammogram, breast angiosarcoma, breast radiology, pseudoangiomatous stromal hyperplasia, male breast cancer, invasive ductal carcinoma, gynecomastia, benign hypoechoic masses

## Abstract

Pseudoangiomatous stromal hyperplasia (PASH) in male patients is a rare condition that represents a hormonally-induced proliferation of mesenchymal tissue of the breast. This benign pathology is often undiagnosed due to many reasons. When PASH presents as a breast mass, it appears innocent, developing as a smooth and well-circumscribed tumor. Furthermore, it does not elicit suspicious findings on imaging. These points often halt further investigation of many breast abnormalities. Breast masses are statistically most likely to be gynecomastia when they arise in men. However, they are important to investigate because, although rare, breast cancer can occur in men. Furthermore, the benign conditions of the breast that commonly affect women can also impact male patients. It is oftentimes overlooked that men too can experience hormonal stimulation of the breast tissue. The following case describes this rare but important instance of a male patient diagnosed with PASH following a previous diagnosis of infiltrative ductal carcinoma in situ of the contralateral breast.

## Introduction

Approaching breast masses in male patients is often deemed unchartered territory, without a well-defined clinical algorithm. Due to physical exam findings being nonspecific, physicians must rely on mammogram and ultrasound for further investigation. These studies reveal specific markers of pathology that suggest either malignancy or a benign condition. Breast cancer commonly presents as a solid, hypoechoic subareolar mass with an irregular shape, which can be spiculated or microlobulated, with ultrasound imaging showing increased vascularity. The more common diagnosis in a male patient includes benign conditions, most frequently gynecomastia. There are three sonographic findings characteristic of gynecomastia - nodular, dendritic, and diffuse glandular. The nodular subtype is a well-circumscribed discoid hypoechoic area, in the retroareolar position. The diffuse glandular subtype appears similar to a normal female breast. Lastly, the dendritic subtype is a flame-shaped density radiating from the nipple, in the subareolar position. This subtype can be mistaken for malignancy and therefore must be followed as such [1].

Pseudoangiomatous stromal hyperplasia (PASH) is defined as a benign proliferation of mesenchymal tissue in the breast. This hyperplasia is an expansion of stromal myofibroblasts, which can be designated microscopically by the expression of cluster of differentiation 34, vimentin, smooth muscle actin, desmin, and B-cell lymphoma 2. On the contrary, endothelial markers, such as S100 or cytokeratin, have not been identified in PASH [2]. Macroscopically, PASH is consistently regarded as a smooth, rubbery, and well-circumscribed tumor, typically without any signs of hemorrhage or necrosis, defined by Powell et al. as nodular or tumoriform PASH [3]. Although, this can also affect individuals surreptitiously, without the emergence of an actual mass. This variant is called the mammary form [4-6].

To reflect on the benign nature of this lesion, it is important to highlight that only one case has been reported to transform into a malignant pathology. Beyond this, PASH is very rarely associated with malignancy or ductal carcinoma in situ [2]. The lack of features concerning for a malignant process does not lessen the importance of making a diagnosis in a tumor of undetermined significance [5]. Reviewing the histology of PASH lesions is critical in differentiating it from the many other causes of breast masses. Two tumors that are particularly similar to the histology of PASH include low-grade angiosarcoma and phyllodes tumors [2]. Even though diagnosing a patient accurately with PASH does not seem clinically relevant due to its harmless nature, it is pivotal in ensuring a patient is not misdiagnosed with low-grade angiosarcoma and treated unnecessarily. The characteristics that are unique to PASH include interanastomosing and angulated slit-like spaces. These spaces are typically lined by thin spindle cells and surrounded by a collagenous stroma. This is where potential for misdiagnosis with low-grade angiosarcoma can occur. These slits are erroneously deemed vascular spaces, despite the lack of red blood cells, but in reality, these slits are due to a fixation artifact. Beyond this, signs of epithelial cell atypia are not present in PASH. The epithelium is generally normal with uniform nuclei and intermittent hyperplasia or duct papillomatosis [5]. It is unclear whether this hyperplasia is an expansion of intralobular stroma, causing a shift of the interlobular stroma, or if the interlobular stroma is growing itself, despite being a hormonally-insensitive tissue [5,7]. The following case demonstrates a male patient with separate benign and malignant conditions of opposite breasts, with an uncommon condition discovered during an abnormally extensive workup.

## Case presentation

A 67-year-old male presented with complaints of a mass in his right subareolar region. He has an extensive past medical history of chronic obstructive pulmonary disease, coronary artery disease, congestive heart failure, hypertension, hyperlipidemia, type two diabetes mellitus, chronic kidney disease, arthritis, and gout, now burdened with several unexpected conditions of the breast. He proceeded to have a diagnostic mammogram upon his original presentation, which demonstrated an oval mass with round calcifications measuring 15 mm in the right subareolar region. These suspicious findings prompted a right breast ultrasound in conjunction with core biopsies. These studies resulted in his diagnosis of infiltrative ductal carcinoma in situ of the right breast, with a Bloom-Richardson grade two out of three, with perineural invasion. With these findings, the decision was made to undergo a mastectomy.

**Figure 1:**
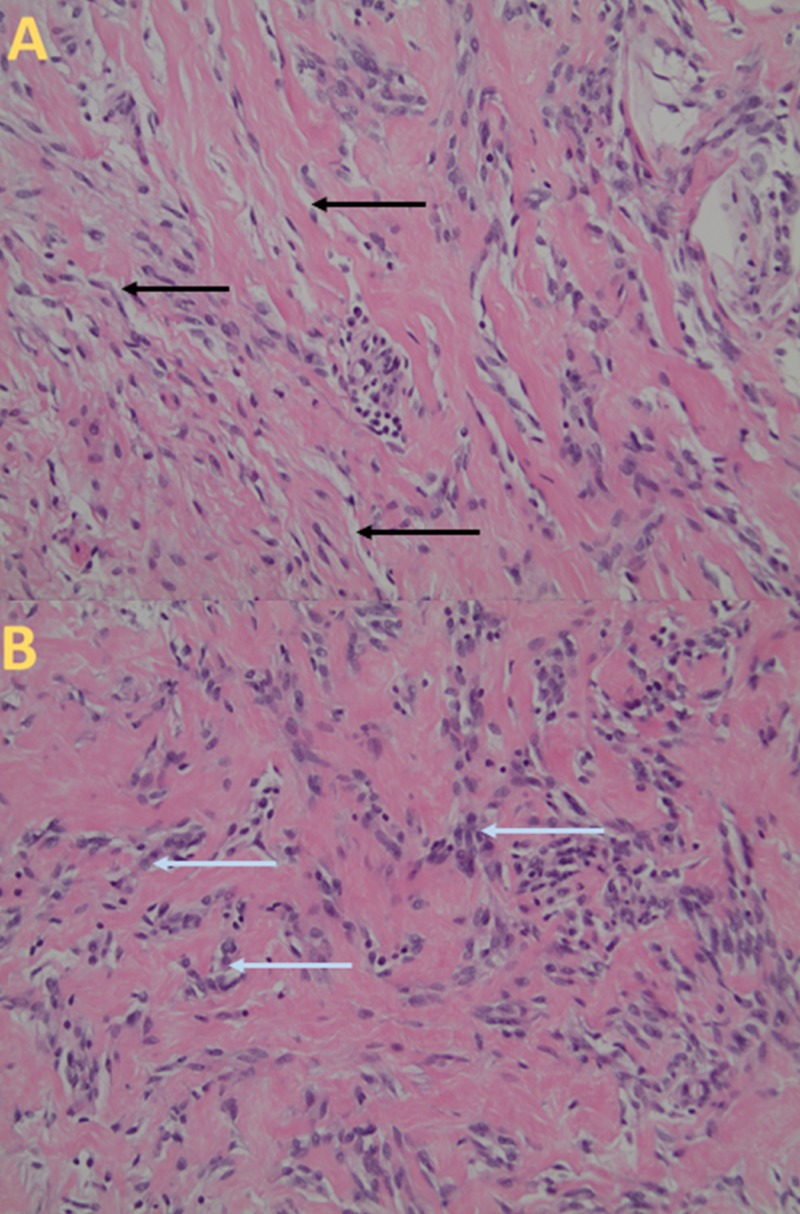
Histologic Appearance of Pseudoangiomatous Stromal Hyperplasia (A) The histopathology sample shows a network of slender corded myofibroblasts, lining narrow inconspicuous white empty spaces (black arrows) with focally dilated spaces at a magnification of 200 times. (B) The histopathology sample shows a complex network of anastomosing spaces lined by myofibroblasts (light blue arrows) separated by collagenized stroma at a magnification of 200 times.

Eleven years later, the patient returned with concern for another mass, this time in his left subareolar region. Subsequent mammogram, ultrasound and core needle biopsies were utilized to assess for recurrence of his infiltrative ductal carcinoma in situ. This workup resulted in a very distinct pathology, PASH (Figure 1).

## Discussion

**Figure 2:**
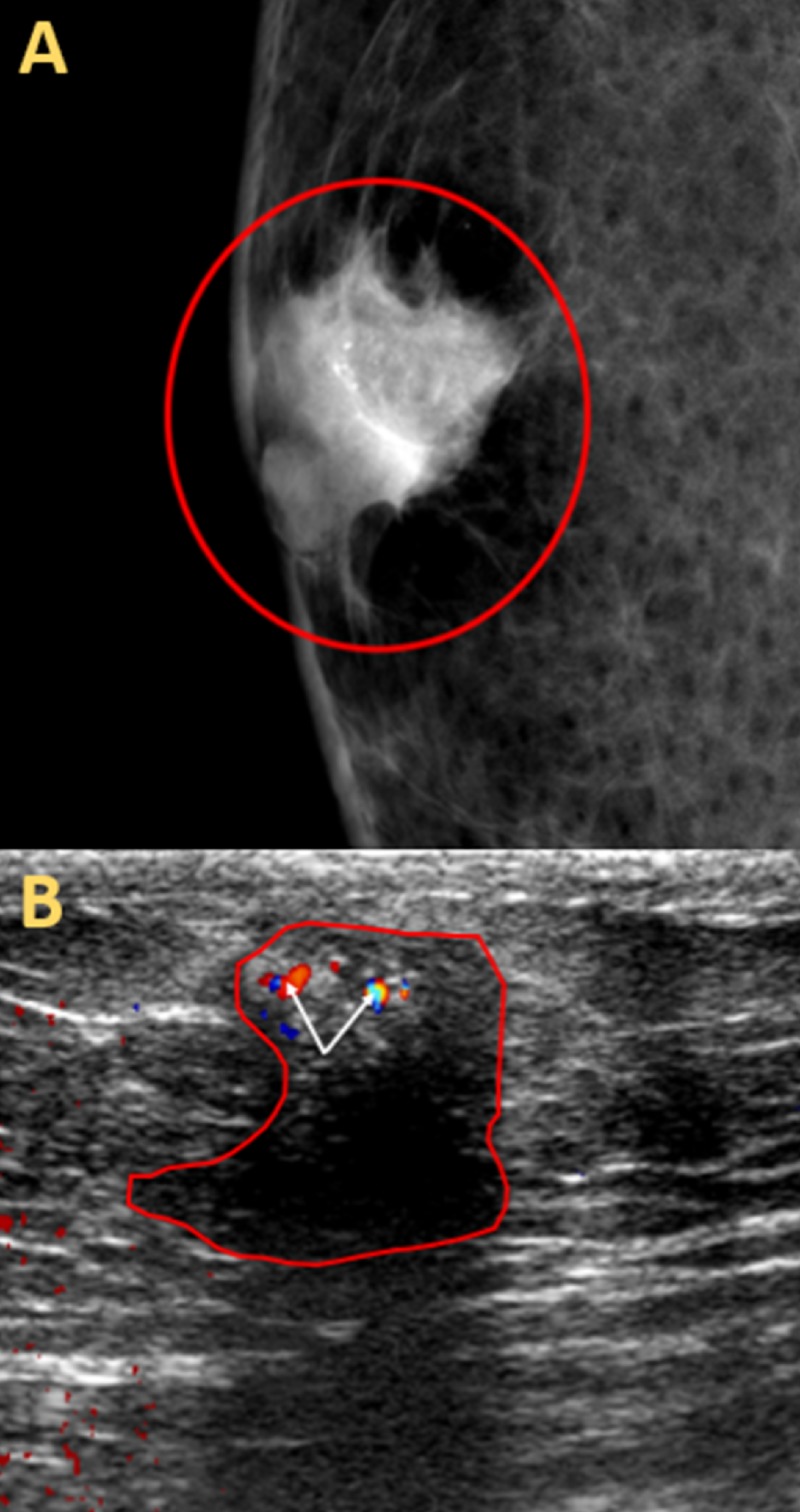
Imaging Findings of Infiltrative Ductal Carcinoma In Situ (A) A diagnostic mammogram of right breast shows an irregular mass with spiculated margins (red circle), measuring 15 millimeters with associated punctate calcifications in the subareolar region. (B) A diagnostic ultrasound of the right breast shows a hypoechoic irregular mass with angular margins (red outline) and internal vascularity (white arrows), measuring 15 millimeters in the subareolar region.

Overall, PASH cases have been scarcely reported since the initial documented case in 1986, by Vuitch et al. [5]. By 2005, there were only 109 total cases of PASH described in the literature, despite the argument that up to 23% of breast biopsies have incidental findings of non-tumor forming PASH [2,4,8]. Hargaden et al. interpreted the mammographic and sonographic features of PASH in 149 patients. Sixty-nine percent of patients in the clinical finding group did not appear to have a mammographic abnormality [9]. The most commonly reported mammographic irregularity in this study was a circumscribed mass, while the sonographic anomaly shared among many of the patients was a well-demarcated, oval, hypoechoic mass [6,9]. These results highlight that there is no specific radiologic manifestation linked to PASH, and that these unsuspicious findings would not typically prompt a physician to obtain a sample for pathology. This leads us to an important point regarding our patient's case. His initial mammogram and ultrasound images were suspicious for cancer, with irregularity of his right breast mass and increased vascularity of the lesion. However, these findings were absent from his left breast imaging eleven years later (Figure 2).

**Figure 3:**
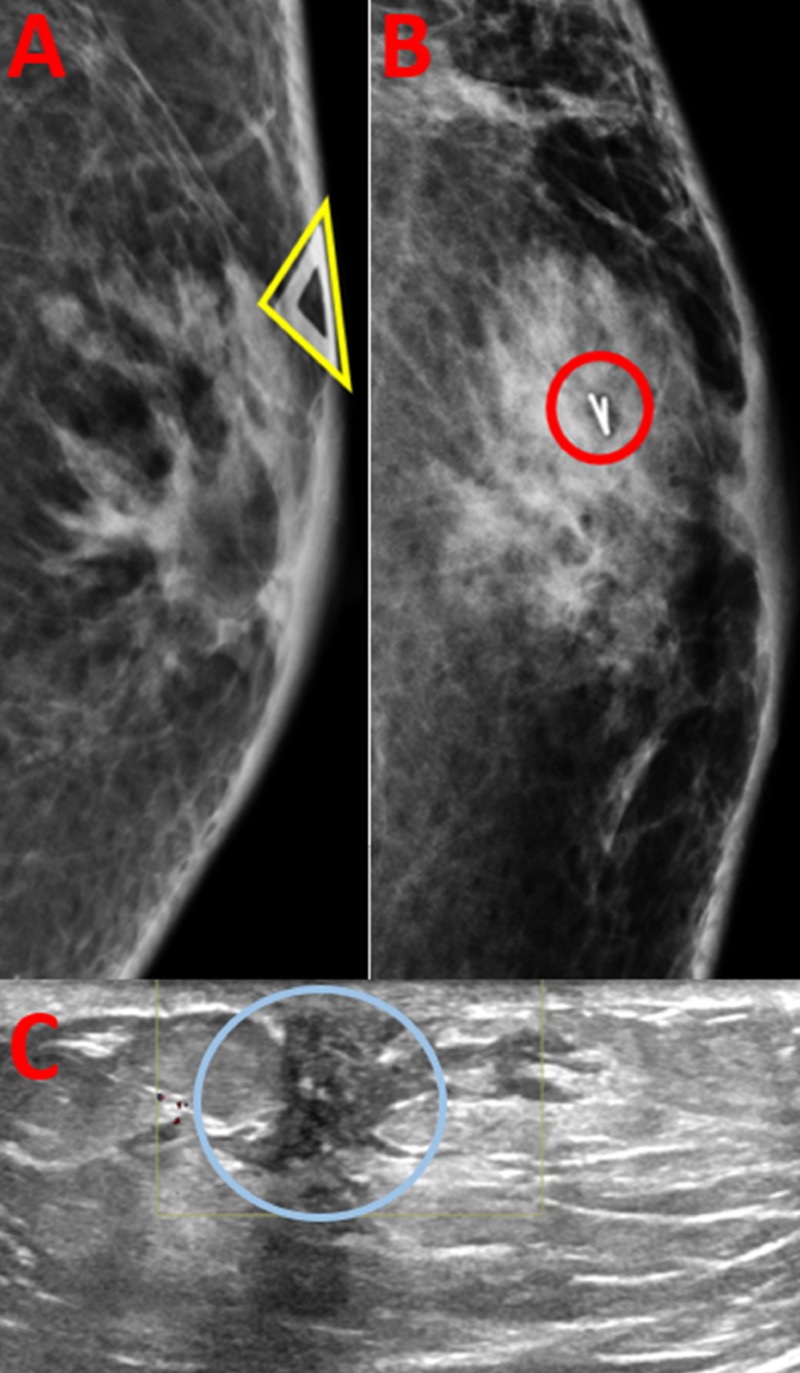
Imaging Findings of Pseudoangiomatous Stromal Hyperplasia (A) A diagnostic mammogram of left breast shows a subareolar focal asymmetry at the site of palpable marker (yellow triangle). (B) A post-biopsy mammogram of left breast demonstrates a ribbon biopsy marker clip within the biopsied mass (red circle), in the subareolar region. (C) A diagnostic ultrasound of left breast shows a hypoechoic irregular mass with angular margins (blue circle) with no internal vascularity, measuring 9 millimeters in the subareolar 12 o’clock region.

The ultrasound imaging for this patient on his second presentation showed a hypoechoic, irregular mass, with angular margins and without internal vascularity. The mass measured 9 mm in the subareolar 12 o'clock region, consistent with nodular-type gynecomastia (Figure 3).

**Figure 4:**
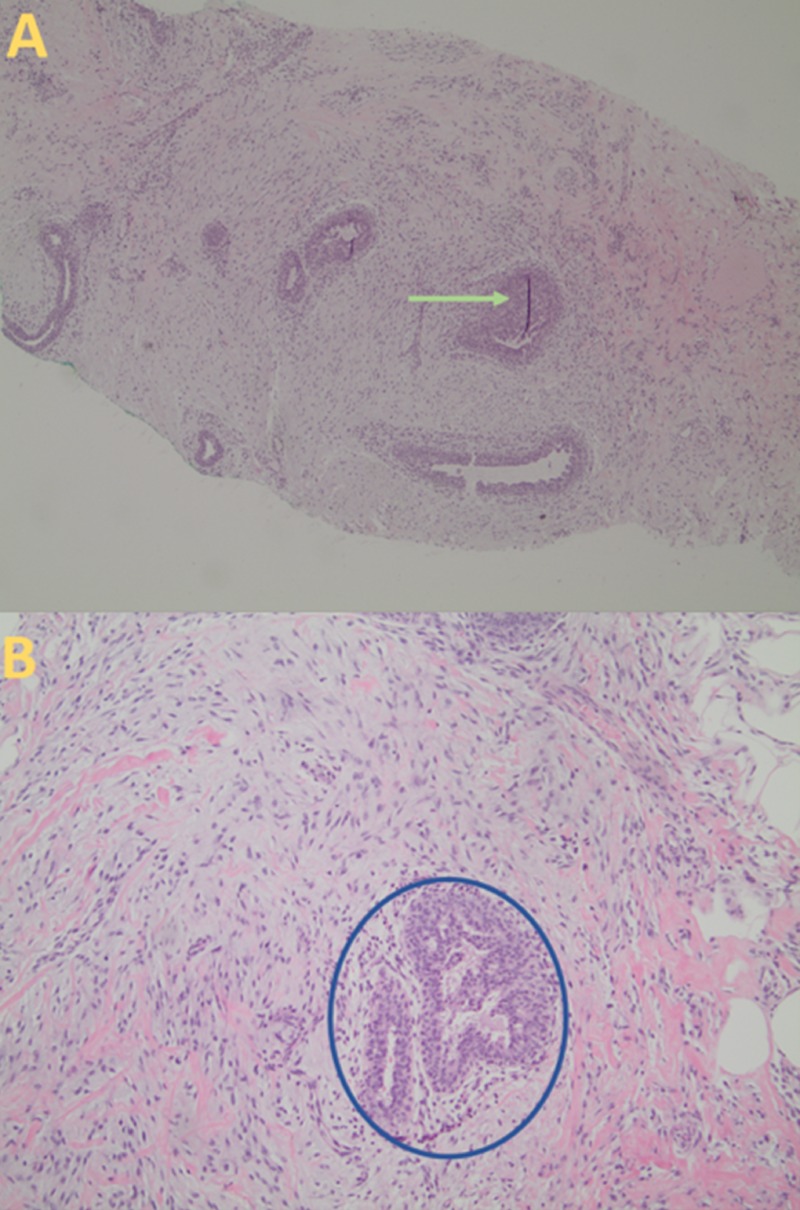
Histological Findings of Gynecomastia (A) An ultrasound-guided core biopsy shows increased stromal cellularity and edema at a magnification of 40 times (green arrow). (B) The histopathology sample demonstrates ductal epithelial hyperplasia with flat or papillary patterns, and increased periductal stromal cellularity (blue circle) at a magnification of 100 times.

This would commonly halt further workup of a patient’s breast mass, but there was motivation for further pursuit of diagnosis via biopsy, due to this patient’s medical history. As expected, this biopsy resembled PASH, in addition to gynecomastia, with increased periductal stromal cellularity, edema, and ductal epithelial hyperplasia with flat or papillary patterns (Figure 4).

It is highly likely that due to his history of right breast cancer, the patient received an abnormally extensive investigation to diagnose a seemingly benign condition.

Due to few indications for biopsying innocent lesions, it may be safe to assume that this condition has overall been underreported. The 149 patients in the study by Hargaden et al. were followed for at least four years, none of which acquired carcinoma at the same site as their PASH diagnosis [9]. Two patients in this study subsequently developed a malignancy of the opposite breast, but overall, there was a 2% recurrence rate of PASH, totaling to three patients out of the 149 studied. This information justifies the argument that PASH is a separate diagnosis and is not a high-risk lesion for cancer [9,10]. The preceding patient discussed strengthens this assertion through the diagnosis of both PASH and infiltrative ductal carcinoma in situ of opposite breasts at separate times.

The patient population affected by PASH speaks to the hypothesized etiology; many of the affected patients are premenopausal or perimenopausal women, which is largely a hormonally-active population [2]. Ferreira et al. reported that 62% of patients in their study were pre-menopausal and had some exposure to hormones, exogenous and/or endogenous [10]. Bowman et al. also discuss two men that were diagnosed with PASH, one who was a transgender male on hormone therapy, and the other with gynecomastia [2]. Gynecomastia in men is typically a result of an underlying medical condition that causes an increase in estrogen levels. This can be seen in cirrhosis and estrogen-producing tumors [4]. The link between gynecomastia and PASH in male patients has been further studied by Badve and Sloane, showing 44 male patients with gynecomastia (47.4%) to have at least one focal point of PASH [7,11]. Reciprocally, it has been determined that 98% of men with PASH display gynecomastia. It is interesting to note that this study further explored the various stages of gynecomastia in relation to this disease, finding a stronger relation between PASH and the early and intermediate phases of gynecomastia.

In 1958, Ozzello and Speer correlated breast histopathologic changes with the differences seen in hyaluronidase-susceptible content of the stroma [5,12]. It was found that this content increased in the premenstrual phase after day 12 to 15, and that the secretory activity of the acini increased during this time as well. Conversely, both hyaluronidase-susceptible content of the stroma and acini secretory activity declined following menstruation. Their argument was that these stromal changes were the reaction of estrogen-primed tissue to progesterone [3,5,9,13]. Additionally, Bowman et al. reports that in their study, 18 out of 19 (95%) samples stained positive for estrogen or progesterone receptors [2]. The association of hormones with PASH is clear. This supports the hypothesis that the abnormal myofibroblast activity is an exaggerated response to hormones [2,14]. Since PASH has been reported to improve with hormonal manipulation or tamoxifen therapy, a selective estrogen receptor modulator, further strengthens this theory [3,9].

Due to the scarcity of PASH diagnoses, surgical excision has been the standard of treatment for many years. The preceding patient also went on to have a surgical excision of his tumoriform PASH. Ferreira et al. discuss that this paradigm is changing, as many cases are now definitively diagnosed via image-guided core needle biopsy [10]. This method of diagnosis allows for close clinical and radiologic surveillance without immediate surgical excision of the mass.

## Conclusions

While PASH has scarcely been reported in the literature, it is even more unique to see this condition described in men. The apparent link between PASH and hormonal stimulation highlights the unusual nature of this case in a male. Prospectively, PASH should be heavily considered in men that experience a significant amount of exposure to estrogen and progesterone, or have known gynecomastia.
